# Clinical implications of interstitial pneumonia with autoimmune features diagnostic criteria in idiopathic pulmonary fibrosis: A case control study

**DOI:** 10.3389/fmed.2023.1087485

**Published:** 2023-02-16

**Authors:** Sara Tomassetti, Claudia Ravaglia, Silvia Puglisi, Athol U. Wells, Jay H. Ryu, Marcello Bosi, Alessandra Dubini, Sara Piciucchi, Francesco Girelli, Paola Parronchi, Federico Lavorini, Elisabetta Rosi, Valentina Luzzi, Marco Matucci Cerinic, Venerino Poletti

**Affiliations:** ^1^Department of Experimental and Clinical Medicine, Careggi University Hospital, Florence, Italy; ^2^Interventional Pulmonology Unit, Careggi University Hospital, Florence, Italy; ^3^Department of Diseases of the Thorax, GB Morgagni Hospital, Forlì, Italy; ^4^ILD Unit, Pulmonary Medicine, Royal Brompton Hospital, London, United Kingdom; ^5^Division of Pulmonary and Critical Care Medicine, Mayo Clinic, Rochester, MN, United States; ^6^Pathology Unit, GB Morgagni Hospital, Forlì, Italy; ^7^Radiology Unit, GB Morgagni Hospital, Forlì, Italy; ^8^Internal Medicine, GB Morgagni Hospital, Forlì, Italy; ^9^Pulmonary Unit, Careggi University Hospital, Florence, Italy; ^10^Department Respiratory Diseases & Allergology, Aarhus University Hospital, Aarhus, Denmark

**Keywords:** idiopathic pulmonary fibrosis, interstitial lung diseases, interstitial pneumonia with autoimmune features, disease behavior, mortality, circulating autoantibodies

## Abstract

**Background:**

A subgroup of IPF patients can meet IPAF criteria (features suggesting an underlying autoimmune process without fulfilling established criteria for a CTD). This study was aimed to evaluate whether IPAF/IPF patients compared to IPF patients differ in clinical profile, prognosis and disease course.

**Methods:**

This is a retrospective, single center, case–control study. We evaluated 360 consecutive IPF patients (Forlì Hospital, between 1/1/2002 and 28/12/2016) and compared characteristics and outcome of IPAF/IPF to IPF.

**Results:**

Twenty-two (6%) patients met IPAF criteria. IPAF/IPF patients compared to IPF were **more frequently females** (*N* = 9/22, 40.9% vs. *N* = 68/338, 20.1%, *p* = 0.02), suffered more frequently from gastroesophageal reflux (54.5% vs. 28.4%, *p* = 0.01), and showed a higher prevalence of **arthralgias** (86.4% vs. 4.8%, *p* < 0.0001), **myalgias** (14.3% vs. 0.3%, *p* = 0.001) and **fever** (18.2% vs. 1.9%, *p* = 0.002). The serologic domain was detected in all cases (the most frequent were ANA in 17 and RF in nine cases) and morphologic domain (histology features) was positive in 6 out of 10 lung biopsies (lymphoid aggregates). Only patients with IPAF/IPF evolved to CTD at follow-up (10/22, 45.5%; six rheumatoid arthritis, one Sjögren’s and three scleroderma). The presence of IPAF was a positive prognostic determinant (HR 0.22, 95% CI 0.08–0.61, *p* = 0.003), whereas the isolated presence of circulating autoantibody did not impact prognosis (HR 1.00, 95% CI 0.67–1.49, *p* = 0.99).

**Conclusion:**

The presence of IPAF criteria in IPF has a major clinical impact correlating with the risk of evolution to full blown-CTD during follow-up and identifying a subgroup of patients with a better prognosis.

## Introduction

In 2015, ATS/ERS international task force defined the criteria describing a new research entity named IPAF (interstitial pneumonia with autoimmune features) (1). This entity identifies patients with an idiopathic interstitial pneumonia (IIP) that have clinical, serologic, or morphologic features suggesting an underlying autoimmune process but do not meet established criteria for a connective tissue disease (CTD) (2). IPAF patients are more frequently female presenting with non-specific interstitial pneumonia (NSIP) pattern in the majority of cases (3, 4). Despite the apparently divergent profile of IPAF compared to idiopathic pulmonary fibrosis (IPF), this association has been described in some retrospective cohorts (3, 5–8).

IPF can be reclassified as IPAF when, in addition to the usual interstitial pneumonia (UIP) pattern, have a combination of one feature from at least two of three different domains; clinical, serologic or morphologic [either pathological (i.e., coexisting histopathology pattern of UIP with interstitial lymphoid aggregates with germinal centers, diffuse lymphoplasmacytic infiltration, and less frequently NSIP/OP overlap) or related to a multi-compartment involvement (i.e., unexplained pleural effusion or thickening, unexplained pericardial effusion or thickening, unexplained intrinsic airway disease, unexplained pulmonary vasculopathy)] (1, 5).

The paucity of studies investigating the impact of IPAF features on IPF natural history provides a strong rationale for the present study that was conducted in a large and well-defined IPF cohort using rigorous inclusion criteria for IPAF and was aimed to evaluate whether IPAF/IPF patients compared to IPF patients have a different prognosis and disease course.

## Materials and methods

### Study design and patient selection

In this single-center, retrospective, investigator initiated comparative study, we evaluated consecutive patients presenting to the pulmonary unit of the GB Morgagni Hospital (Forlì, Italy) with suspected interstitial lung disease who received a multidisciplinary diagnosis of IPF (between January 1, 2002, and December 28, 2016). Patients with incomplete clinical data, less than 3 months of follow-up and those without a complete autoimmune clinical and serological evaluation performed at our center were excluded. Baseline and follow-up data were collected as detailed in the Supplementary material, p. 2. Given the wide time spam of diagnosis, all IPF diagnosis were reviewed based on ERS/ATS 2018 criteria. Criteria for IPAF inclusion followed the ERS/ATS 2015 statement, details are reported in the Supplementary material, p. 3 (2).

This study was approved by the Comitato Etico di area vasta ROMagna, Italy (CEROM approval: protocol number 30/2020 I.5/284).

### Outcomes

The primary endpoint was the prognostic significance of the presence of IPAF among IPF patients. This was measured by comparing transplant-free survival for IPF with IPAF to that of IPF without IPAF.

Secondary endpoints included:

(1) The prognostic significance of the presence of positive autoimmune serology alone (i.e., without IPAF) in IPF patients, compared to IPAF/IPF and to IPF only (i.e., no positive serology and no IPAF). This was measured comparing transplant-free survival for IPF with positive autoimmune serology to that of IPF with IPAF and to that of IPF only.
(2) Evaluation of natural history: development of full-blown CTD at follow-up. We described the baseline characteristics and compared the prevalence of CTD at follow-up between three patients subgroups: IPF with IPAF, IPF with positive autoimmune serology alone (i.e., without IPAF) and IPF only (i.e., no positive serology and no IPAF).

### Statistical methods

For baseline data, the summary descriptive statistics were be generated with categorical data displayed as absolute numbers and relative frequencies. Continuous data were shown as mean (SD) for normally distributed data or as median (interquartile range) for skewed distribution. Comparisons between groups was performed using a *t*-test or Chi^2^ test, as appropriate. We used exact probability values (*p* values) and an alpha level of 0.05.

For regression analysis, sample size calculation met the rule of thumb of at least 10 observation per variable, with 170 observed events we could evaluate 17 variables without overfitting. The small size of missing data allowed an analysis restricted to individuals with complete information on all variables of the main analysis (complete case analysis). The fundamental method of univariable/multivariable analysis was Cox regression. Causal model based on previous literature was used to identify confounders: age, gender, smoking status, comorbidities and disease severity. The models were formulated by systematically removing predictors that were not significant using a backward selection procedure removing variables with *p*-values ≥0.2 and excluding covariates with significant collinearity (*r* > 0.8) at univariate analysis. The proportional hazard assumption for each predictor was tested using approximate score statistic of linear correlation between the rank order of failure times in the sample and Schoenfeld partial residuals. We calculated hazard ratios (HRs) for overall mortality analyses. Patients were censored at death, lung transplant, or date of last known follow-up. Data cut-off was December 28, 2016. Results are reported as HRs, 95% CIs and *p* values, and are shown graphically as Kaplan–Meier survival curves. All statistical analyses were performed using STATA 15.

## Results

### Population

We extracted from the Forlì database 703 consecutive IPF patients, between January 1, 2002 and December 28, 2016. Among those 360 met protocol requirements and were included in the study. A vast minority (*N* = 274) was excluded due to the lack of rheumatologic and/or serologic evaluation performed at our center. The flow chart of cases inclusion is presented in the Supplementary Figure S1 (p. 4). Clinical characteristics of included and excluded cases were similar, but the prognosis was worse in the included group of patients compared to excluded (HR adjusted for age, gender, smoke, %FVC, %DLco and lung cancer was 1.39, 95%CI 1.11–1.73, *p* = 0.003), as detailed in the Supplementary material, p. 5–7. Twenty-two (6%) patients met IPAF criteria (IPF/IPAF cases). Among the remaining 338 IPF cases that did not meet IPAF criteria, 43 (12% of the total) showed isolated autoimmune serology positivity lacking other positive domains. Characteristics of patients are reported in Table 1.

**Table 1:** Patient characteristics and comparison between IPAF/IPF and non-IPAF/IPF.

	Total cohort	IPAF/IPF	non-IPAF/IPF	Non-IPAF/IPF		*p*-val*
				Positive serology	Negative serology	
Sample size, *N*	360	22	338	43	295	
Female sex, *N* (%)	77 (21.4)	9 (40.9)	68 (20.1)	12 (27.9)	56 (19.0)	**0.020**
Age, median (IQR)	66.4 (8.53)	66.5 (8.55)	64.4 (8.08)	64.9 (7.3)	66.7 (8.7)	0.200
Current or former smokers, *N* (%)	255 (71.1%)	14 (63.6)	241 (71.5)	32 (76.2)	209 (70.8)	0.430
Family history of ILDs, *N* (%)	58 (16.1)	1 (4.5)	57 (16.9%)	7 (16.3)	50 (16.9)	0.130
Patients with comorbidities, *N* (%)	279 (77.5)	19 (86.4)	260 (76.9%)	30 (69.8)	230 (78.0)	0.300
N of comorbidities, median (range)	1.15 (0.90)	1.40 (1.14)	1.14 (0.89)	0.97 (0.83)	1.16 (0.89)	0.100
Lung cancer, *N* (%)	33 (9.2)	1 (4.5)	32 (9.5)	6 (14.0)	26 (8.8)	0.400
Pulmonary hypertension, *N* (%)	117 (39.9)	6 (31.6)	111 (40.5%)	15 (41.7)	96 (40.3)	0.400
GERD, *N* (%)	107 (30.1)	12 (54.5)	95 (28.4%)	17 (39.5)	78 (26.8)	**0.010**
% of pred. FVC, mean (SD)	79.64 (18.98)	86.72 (14.25)	79.18 (19.18)	78.53 (22.13)	79.27 (18.75)	0.070
% of pred. DLco, mean (SD)	52.53 (17.23)	59.41 (16.96)	52.08 (17.18)	58.78 (15.57)	51.83 (17.40)	**0.050**
GAP stage						
GAP stage I, *N* (%)	249 (69.5)	18 (81.8)	231 (68.8%)	26 (60.5)	205 (70.0)	0.200
GAP stage II, *N* (%)	100 (27.9)	4 (18.2)	96 (28.6%)	15 (34.9)	81 (27.6)	0.290
GAP stage III, *N* (%)	9 (2.5)	0	9 (2.7%)	2 (4.7)	7 (2.4)	0.440
Symptoms onset^						
Acute, *N* (%)	16 (5.2)	2 (10)	14 (4.9)	2 (5.0)	12 (4.9)	0.330
Subacute, *N* (%)	41 (13.5)	3 (15)	38 (13.4%)	7 (17.5)	31 (12.7)	0.840
Chronic, *N* (%)	247 (81.2)	15 (75)	232 (81.7%)	31 (77.5)	201 (82.4)	0.460
Symptoms						
Cough, *N* (%)	204 (60.9)	13 (59.1)	191 (61.0%)	26 (60.5)	165 (61.1)	0.860
Dyspnea, *N* (%)	308 (91.9)	19 (86.4)	289 (92.3%)	42 (97.7)	247 (91.5)	0.320
Arthralgias, *N* (%)	34 (10.1)	19 (86.4)	15 (4.8%)	10 (23.3)	5 (1.9)	**<0.0001**
Myalgias, *N* (%)	4 (1.2)	3 (14.3)	1 (0.3)	1 (2.3)	0 (0.0)	**0.001**
Recurrent fever, *N* (%)	10 (2.9)	4 (18.2)	6 (1.9)	1 (2.3)	5 (1.9)	**0.002**
Weight Loss, *N* (%)	2 (0.6)	0 (0.0)	2 (0.6)	0	2 (0.7)	0.710
Signs						
Velcro, *N* (%)	299 (90.6)	20 (95.2)	279 (90.3)	40 (93.0)	239 (89.8)	0.450
Digital clubbing, *N* (%)	32 (9.7)	3 (14.3)	29 (9.4)	10 (23.3)	19 (7.1)	0.460
Progression to full-blown CTD	10 (2.7)	10 (45.5)	0	0	0	**<0.0001**

Values are expressed as mean (SD), median (IQRs), numbers (column %). Statistically significant *p*-values are reported in bold.

* *p*-Value comparing IPAF/IPF to non-IPAF/IPF.

^ Acute onset: < 1 month, Subacute onset < 6 months, chronic onset > 6 months.

### IPAF cases compared to non-IPAF cases

Comparison between IPAF/IPF patients and non-IPAF/IPF patients is shown in Table 1. IPAF/IPF patients compared to non-IPAF/IPF were **more frequently females** (*N* = 9/22, 40.9% compared to *N* = 68/338, 20.1%), *p* = 0.02. No significant differences were noted in age, smoking history, family history and comorbidities profile with the notable exception of **GERD** that was significantly more prevalent in IPAF/IPF compared to non-IPAF/IPF (54.5% compared to 28.4%, *p* = 0.01). The pulmonary function profile showed **a slight but significantly higher DLco** in the IPAF/IPF patients (59.41 vs. 52.8, *p* = 0.05) and a marginally higher FVC not statistically significant (86.72 vs. 79.18, *p* = 0.07) without significant differences in the GAP stage distribution. Similarly to non-IPAF/IPF patients, IPAF/IPF patients presented in the vast majority with a chronic onset (75%) of dyspnea (86.4%) and/or cough (59.1%). However, in contrast to what observed in non-IPAF/IPF patients, all IPAF/IPF patients presented with at least one systemic symptom, with a strikingly higher prevalence of **arthralgias** (86.4% vs. 4.8%, *p* < 0.0001), **myalgias** (14.3% vs. 0.3%, *p* = 0.001) and **fever** (18.2% vs. 1.9%, *p* = 0.002) compared to non-IPAF/IPF. Further rheumatologic evaluation of arthralgias revealed inflammatory arthritis only in IPAF/IPF cases, as shown in Table 2.

**Table 2:** IPAF/IPF cases characteristics.

ID	Age	Sex	Latency time from IPF to IPAF	IPAF diagnostic domains				Latency time from IPAF to CTD	CTD at FUP	Treatment
				Serologic domain	Clinical domain	Lung biopsy	Morphologic Domain			
1	43	M	Concurrent	ANA >1:160 nucleolar		TBLC and SLB	Interstitial lymphoid aggregates with germinal centers (UIP)			CYC; Esbriet
2	74	F	2 years	RF; ANTI-CCP; ANA>1/320	Raynaud’s phenomenon			22 months	Rheumatoid Arthritis	Nintedanib; Leflunomide
3	67	F	9 years	ANA >1:640 nucleolar; (Anti-TG)	Inflammatory arthritis and polyarticular morning joint stiffness ≥60 min	TBLC	UIP			Prednisone
4	73	M	4 years	ENA (anti Ro 52)	Inflammatory arthritis; Distal digital tip ulceration (sicca syndrome)			2 years	Sjogren	Pirfenidone; Nintedanib
5	75	F	4 years	ANA>1:320; ANTI-CEMP-B	Inflammatory arthritis (and recurrent low grade fever)					Azathioprine, NAC, Prednisone
6	75	F	Concurrent	(ANA<1:80); RF; ANTI-CCP > 340	Inflammatory arthritis					Nintedanib
7	61	M	4 years	ANA>1:160 nucleolar	Inflammatory arthritis (sicca syndrome)	TBLC	Interstitial lymphoid aggregates with germinal centers (UIP)			Esbriet
8	57	F	Concurrent	ANA>1/320; ANTI-CEMP-B	Inflammatory arthritis	TBLC	Interstitial lymphoid aggregates with germinal centers (UIP)	1 year	Scleroderma	Prednisone, Ciclosporine, RTX
9	64	F	5 years	ANA >1:320 speckled	Inflammatory arthritis					Untreated
10	78	F	3 years	ANA >1:640	Inflammatory arthritis and polyarticular morning joint stiffness ⩾60 min					Esbriet
11	63	M	3 years	RF; ANTI-CCP; ANA>1/320	Inflammatory arthritis					Prednisone
12	70	M	Concurrent	ANA>1:320; RF; ANTI-CCP	Inflammatory arthritis			2 years	Rheumatoid Arthritis	Esbriet; Nintedanib
13	69	M	1 year	ANA>1:320; RF	Inflammatory arthritis	SLB	Interstitial lymphoid aggregates with germinal centers (UIP and few giant cells)			Nintedanib
14	76	F	6 years	ANA 1/640 omogeneo	Inflammatory arthritis and polyarticular morning joint stiffness ≥60 min; myalgias	TBLC	UIP	2 years	Rheumatoid Arthritis	Azathioprine, NAC, Prednisone
15	53	M	2 years	RF; ANTI-CCP	Inflammatory arthritis	SLB	Interstitial lymphoid aggregates with germinal centers (UIP)	1 year	Rheumatoid Arthritis	Esbriet; Prednisone
16	68	M	2 years	ANA >1/320	Inflammatory arthritis; Raynaud phenomenon			7 months	Scleroderma	Pirfenidone
17	72	M	Concurrent	ANA >1/320	Inflammatory arthritis					Prednisone
18	72	M	1 year	ANA >1/320; antiCCP, RF	Inflammatory arthritis	SLB	UIP	6 months	Rheumatoid Arthritis	Prednisone; Leflunomide; CYC
19	76	F	3 years	ANA>1:320; RF; (Anti-TPO)		TBLC	Interstitial lymphoid aggregates with germinal centers (UIP)			Esbriet; Nintedanib, MMF, prednisone
20	59	M	Concurrent	ANA>1:320; ANCA-MPO	Raynaud’s phenomenon			2 years	Scleroderma	Prednisone
21	39	F	Concurrent	ENA: Anti-Ro (SS-A)	Inflammatory arthritis	SLB	UIP			Prednisone
22	64	M	Concurrent	RF; ANTI-CCP; ANCA-MPO	Inflammatory arthritis and polyarticular morning joint stiffness ≥60 min			5 years	Rheumatoid Arthritis	Pirfenidone; Prednisone, Leflunomide

### Non-IPAF/IPF: Comparison between cases with and without circulating autoantibodies

Among the 338 IPF patients that did not meet IPAF criteria, 43 (12.7%) showed a positive autoimmune serology.

**All cases showed isolated positivity** for a single class of autoantibodies. Four patients (4/43, 9%) showed ANCA positivity and one of them developed a full-blown vasculitis after 10 years of follow-up (familial form of IPF with first degree relatives affected by both IPF and CTD-related ILDs). Seventeen (39%) patients had an isolated anti-thyroid positive autoimmunity, all were clinically identified as autoimmune thyroiditis without evidence of systemic autoimmune disease. 11 (11/43, 25%) patients showed isolated ANA positivity, two ENA, two anti-CCP, and 7 RF positivity. None of them developed CTD at follow-up.

**Comparison** of clinical characteristics of non-IPAF/IPF cases with and without positive autoimmune serology are reported in Table 1. There were no statistically significant differences in the clinical and functional profile of the two subgroups (*p* values >0.05, not shown) with two notable exceptions: (1) **higher prevalence of arthralgias in the non-IPAF subgroup with positive** autoantibodies (*N* = 10, 23%) compared to non-IPAF with negative autoantibodies (*N* = 5, 1.9%), *p* < 0.0001. Those patients were not classified as IPAF because arthralgias was interpreted by the rheumatologist as non-specific. (2) **higher prevalence of digital clubbing** in the non-IPAF subgroup with positive autoantibodies (*N* = 10, 23%) compared to non-IPAF with negative autoantibodies (*N* = 19, 7.1%), *p* = 0.003.

### Survival analysis

#### Primary outcome: The prognostic significance of the presence of IPAF among IPF patients

**Figure 1:**
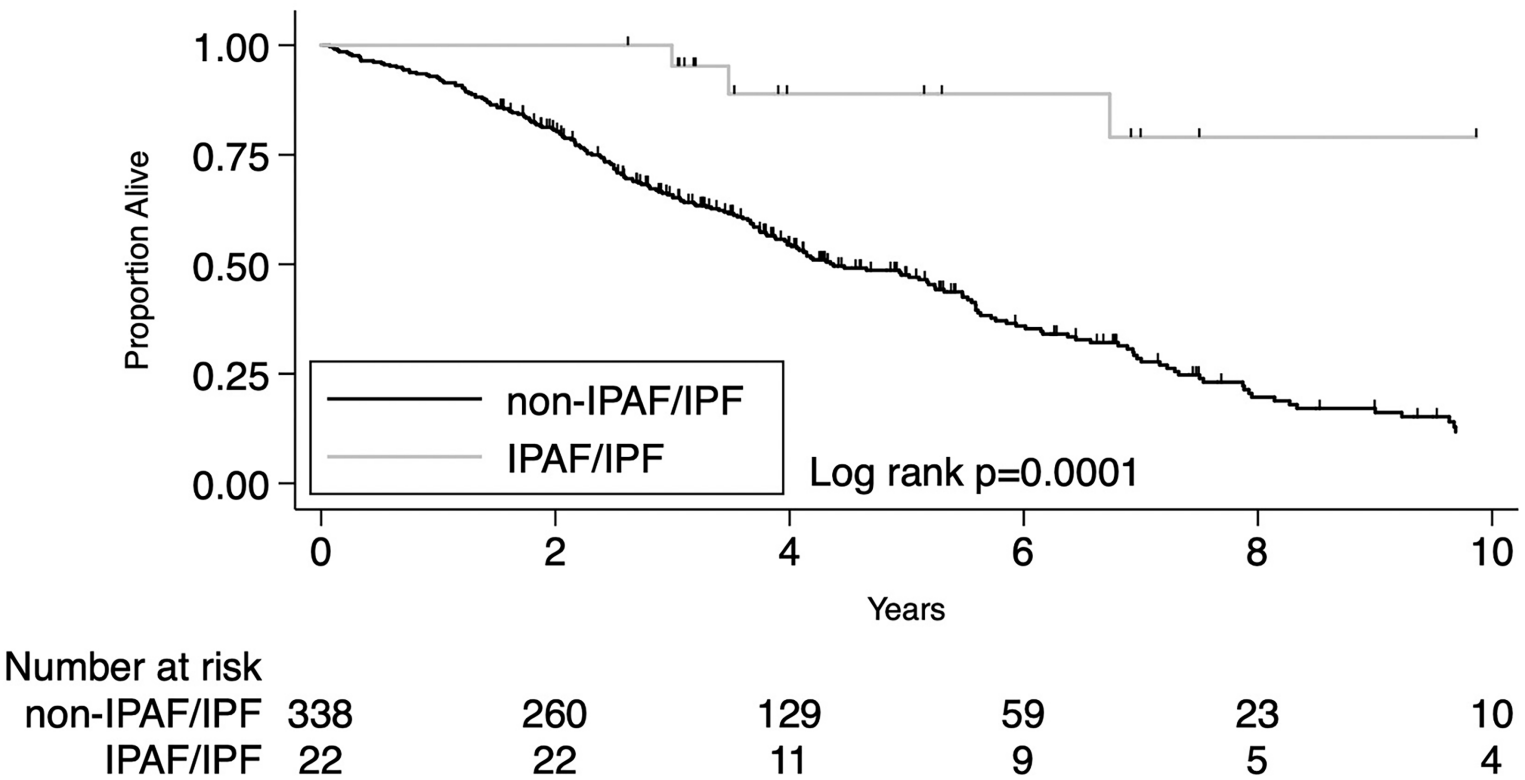
Primary outcome: KM plot for IPF diagnosis meeting IPAF criteria compared to IPF not meeting IPAF criteria.

The presence of IPAF criteria in patients who received a multidisciplinary diagnosis of IPF was associated with significantly lower overall mortality compared to IPF patients lacking IPAF criteria. Despite the small number of cases (*N* = 22), the difference was robust both by univariate analysis HR 0.17 (95% CI 0.06–0.46, *p* < 0.0001) and after adjusting for age, sex, lung cancer, pulmonary function variables (%pred FVC and %pred DLco) and diagnosis before or after 2011, HR 0.22 (95% CI 0.08–0.61, *p* = 0.003). Univariate and multivariate analyses are reported in Table 3. Beside the presence of IPAF criteria the other variables significantly associated with a different prognosis were age, pulmonary function (i.e., % of predicted FVC and % of predicted DLco) and the presence of lung cancer. Median follow-up time was 4.53 years for IPAF/IPF (IQR 3.17–7.50) and 3.39 years for Non-IPAF/IPF (IQR 2.06–5.12). Overall mortality rate per 100 person-year and survival at 1, 3, and 5 years were all significantly different between the two groups, data are reported in Table 4. Figure 1 shows the Kaplan–Meier plot for transplant-free survival of IPAF/IPF and non-IPAF/IPF cases.

**Table 3:** Univariate and multivariate transplant-free survival analysis comparing IPAF/IPF to non-IPAF/IPF total number of cases IPAF/IPF *n* = 22, non-IPAF/IPF *N* = 338.

	Univariate analysis		Multivariate analysis	
	HR (95% CI)	*p*-Value	HR (95% CI)	*p*-Value
Age	1.03 (1.01–1.05)	**0.002**	1.03 (1.01–1.05)	**0.001**
Sex Female	0.90 (0.65–1.26)	0.55	1.00 (0.71–1.40)	0.98
Smoking history	1.20 (0.88–1.63)	0.25	–	–
Comorbidities (yes/no)	0.91 (0.67–1.24)	0.56	–	–
Number of comorbidities	1.07 (0.92–1.24)	0.40	–	–
Lung cancer (yes/no)	1.94 (1.32–2.85)	**0.001**	2.61 (1.72–3.97)	**<0.0001**
Gastroesophageal reflux	0.94 (0.70–1.26)	0.66	–	–
Pulmonary function				
% pred FVC	0.98 (0.97–0.98)	**<0.0001**	0.99 (0.98–1.00)	**0.002**
% pred DLco	0.97 (0.96–0.97)	**<0.0001**	0.97 (0.96–0.98)	**<0.0001**
Diagnostic period (before or after 2011)	0.56 (0.40–0.79)	**0.001**	0.58 (0.40–0.83)	**0.003**
Diagnosis of IPAF/IPF	0.17 (0.06–0.46)	**<0.0001**	0.22 (0.08–0.61)	**0.003**

Statistically significant *p*-values are reported in bold. IPF, idiopathic pulmonary fibrosis; IPAF, interstitial pneumonia with autoimmune features; FVC, forced vital capacity; DLco, carbon monoxide diffusing capacity.

**Table 4:** Survival analysis for the primary outcome of the study: IPAF/IPF compared to non-IPAF/IPF and for the secondary outcome of the study: non-IPAF/IPF with positive autoimmune serology compared to non-IPAF/IPF without positive autoimmune serology.

	Primary outcome		Secondary outcome: non-IPAF/IPF	
	IPAF/IPF	Non-IPAF/IPF	Positive serology	Negative serology
Sample size	22	338	43	295
Number of deaths	4	197	30	167
Number of lung transplants	0	16	2	14
Median time of follow up in years (IQRs)	4.53 (3.15–7.50)	3.39 (2.06–5.12)	3.88 (2.03–5.85)	3.29 (2.07–4.96)
Mortality rate per 100 py	3.13 (1.17–8.34)	16.26 (14.21–18.59)	17.42 (12.32–24.63)	16.06 (13.89–18.29)
Transplant free survival				
At 1 year	100%	92% (95% CI 0.89–0.94)	93% (95% CI 0.79–0.97)	93% (95% CI 0.88–0.95)
At 3 years	95% (95% CI 0.71–0.99)	65% (95% CI 0.60–0.70)	66% (95% CI 0.50–0.78)	65% (95% CI 0.59–0.70)
At 5 years	89% (95% CI 0.63–0.97)	47% (95% CI 0.41–0.53)	51% (95% CI 0.34–0.65)	46% (95% CI 0.40–0.52)

#### Secondary outcome: The prognostic significance of the sole presence of positive autoimmune serology

##### Comparison between IPF with positive serology and IPF only

**Figure 2:**
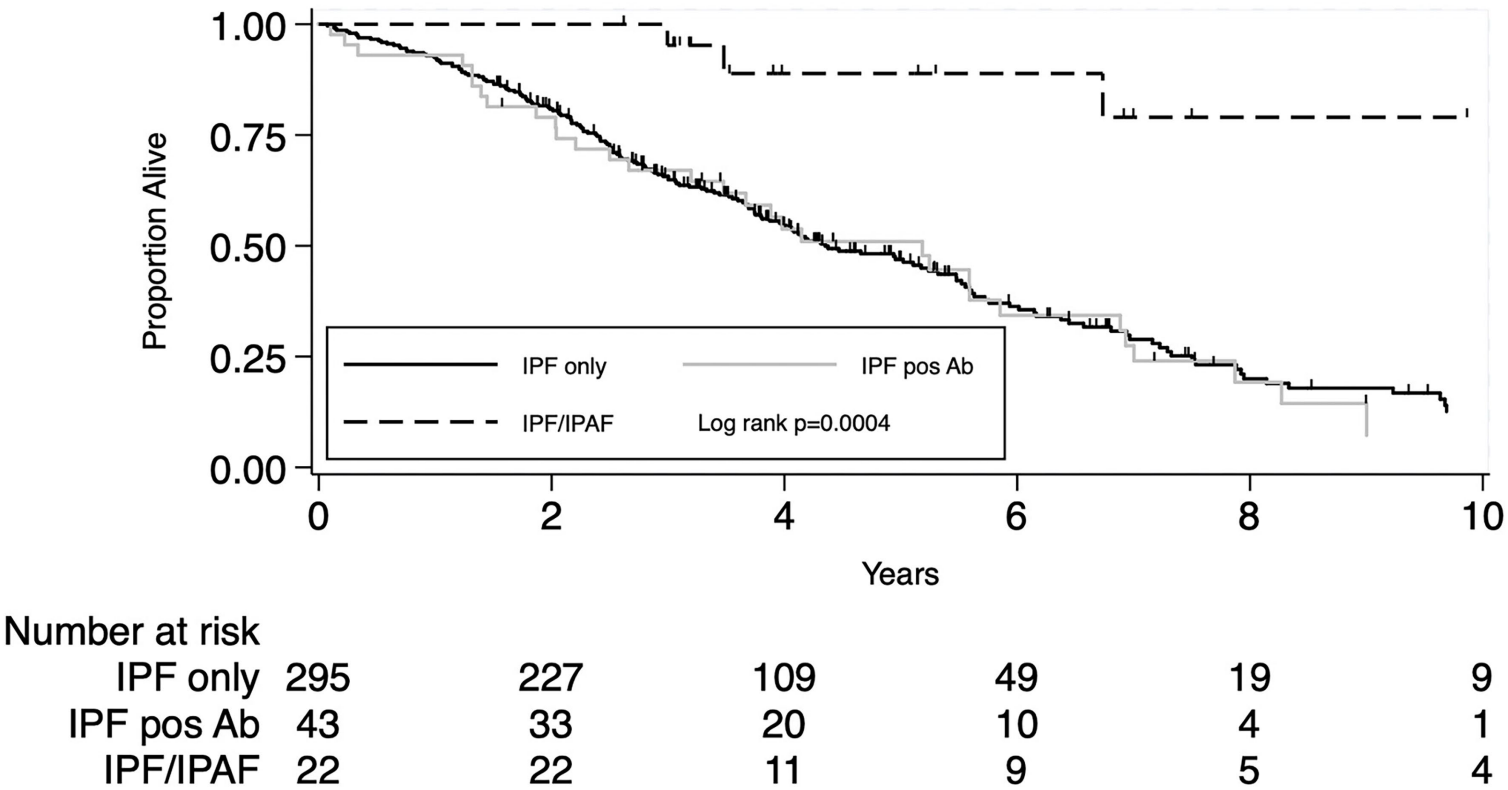
Secondary outcome: KM plot for IPF diagnosis meeting IPAF criteria compared to IPF not meeting IPAF criteria with and without positive autoimmune serology.

When analysis was confined to IPF patients not meeting IPAF criteria (*N* = 338) the presence of positive autoimmune serology (*N* = 43) did not impact patients’ prognosis, unadjusted HR 1.01 (95% CI 0.69–1.48), *p* = 0.96 and HR 1.00 (95% CI 0.67–1.49), *p* = 0.99 after adjusting for age, sex, lung cancer and pulmonary function variables (%pred FVC and %pred DLco). Overall mortality rate per 100 person-year and survival at 1, 3, and 5 years were all similar between the two groups, data are reported in Table 4. Figure 2 shows the Kaplan–Meier plot for transplant-free survival of IPAF/IPF and non-IPAF/IPF cases with and without positive autoimmune serology.

#### Secondary outcome: The evolution to full blown connective tissue disease

Only IPF cases with IPAF features developed full-blown CTD at follow-up. Among IPAF/IPF cases 10 patients (10/22, 45.5%) developed CTD: rheumatoid arthritis (6/22, 27%), scleroderma (3/22, 14%) and one Sjogren. Mean latency time from IPAF diagnosis to CTD diagnosis was 21.5 months (range 6–60 months).

The characteristics of the 22 IPAF cases are reported in Table 2. Fourteen patients were treated with low doses of prednisone and two of them with triple therapy (azathioprine, n-acetyl-cysteine and prednisone). All of them antedates the publication of the PANTHER trial. (9) All 10 IPAF/IPF patients diagnosed after the year 2012 (when antifibrotics became available at our center) were treated with antifibrotics and continued antifibrotic therapy after IPAF diagnosis. Among those, five patients developed CTD (four rheumatoid arthritis and one scleroderma): three of them continued antifibrotics only and two switched to immunosuppressive treatment only. Only one patient diagnosed with IPAF/IPF, that has never developed a clear CTD, is currently treated with the combination of immunosuppressant (prednisone and mofetil-mycophenolate) and nintedanib due to disease progression.

## Discussion

To the best of our knowledge this is the first study evaluating IPAF features and clinical meaning in a well-defined cohort of IPF patients, showing that IPF patients can present IPAF features in a minority of cases (6%) with a clinical profile and natural history strikingly divergent from that of IPF. IPAF/IPF compared to IPF is characterized by:

(1) a significantly better prognosis;
(2) a high risk of evolution to full blown CTD (45.5%), that among IPF patients seems exclusive of those presenting with IPAF features;
(3) a specific clinical profile with a higher prevalence of females and gastroesophageal reflux. Most notably, systemic signs and symptoms, rare in IPF, are universally present in IPAF/IPF (inflammatory arthropathy, myalgias and fever) and are associated with at least one positive autoimmune finding on serology (most commonly elevated levels of ANA and/or RF).

To define IPAF in this cohort of IPF patients we have meticulously applied the current IPAF ERS/ATS criteria to the clinical and serological features. The morphologic domain was defined by histopathology patterns (by surgical or transbronchial cryobiopsy) of UIP with interstitial lymphoid aggregates with germinal centers and/or diffuse lymphoplasmacytic infiltration (with or without lymphoid follicles), and these features were present in one third of IPF/IPAF cases (6/22). The multi-compartment involvement was defined using more stringent criteria: unexplained pleural or pericardial effusion or thickening, or by the presence of unexplained airway disease as seen by histopathology (follicular bronchiolitis or constrictive bronchiolitis). Interestingly none of our IPF/IPAF patients met the multi-compartment features following these restricted criteria. This punctilious approach led to a prevalence of IPAF among IPF cases lower than previously described, but with a strikingly prognostic divergence that previous studies could not detect (5).

We show that the presence of IPAF criteria is a strong positive prognostic determinant (overall mortality HR 0.22, 95% CI 0.08–0.61, *p* = 0.003). Age, lung cancer, pulmonary function (% of pred FVC and % of pred DLco), diagnostic time period (before/after 2011) and IPAF were all significant prognostic factors at both univariate and multivariate survival analysis. IPAF/IPF patients compared to IPF had a significantly lower overall mortality rate per 100 persons year (3.13 vs. 16.26) and a significantly better prognosis (survival at 3 and 5 years 95% vs. 65 and 89% vs. 47%, respectively). Previous studies have compared the prognosis of IPAF to that of historical IPF patients cohorts, showing that IPAF carries a better prognosis compared to IPF (4, 10). However, in those studies patients with IPAF/IPF were either not included (4) or mixed with a majority of non-IPF (NSIP/OP) cases (10). The present study does not include all types of IPAF, it rather focus on patients initially classified as having IPF. Notably no cases were having suspected CTD-ILD. We believe that this is a strength of this study, because here we highlight for the first time that IPAF reclassification can be clinically relevant when a diagnosis of IPF has been made, having prognostic implications that may potentially alter management.

The Oldham study (5) is the most solid study that compared IPAF/IPF (defined by Oldham as IPAF with UIP) to IPF reporting a prevalence of IPAF among IPF patients significantly divergent to what we report in this study (18% vs. 6%), but without survival differences. A possible reason for this discrepancy is the divergent profile of our IPAF/IPF population compared to that of Oldham et al. In our study all but two patients met clinical and serological domain criteria (90%, compared to 6.1% of the Oldham study). Including pulmonary hypertension and FVC/DLco ratios above 1.6 in the definition of multi-compartment criteria, Oldham et al. may have included a higher number of advanced IPF patients with PH in the IPAF group, those patients having selectively poor outcomes may explain the lack of outcome difference observed in that study. In line with this hypothesis is the observation that the presence of multicompartment features can increase the overall mortality risk (HR 2.1, 95% CI 1.19–3.38, *p* = 0.009) (5).

In the absence of IPAF criteria the sole presence of autoantibodies in IPF did not influence survival. It is difficult to compare our results with the existing literature on this topic because our study was not powered to detect the prognostic significance of specific antibodies. In the literature the effect of ANA and RF on all causes mortality does not seem significant. (11) The subsequent development of CTD has been shown to occur in 2.5% of patients previously diagnosed with idiopathic interstitial pneumonia and is in line with the prevalence here observed (10/360, 2.7%) (5, 11, 12). The nice overlap between our data and older studies may suggest that we are evaluating similar cohorts of patients, but with the novel notion that adding IPAF criteria to the simple detection of circulating autoantibodies can significantly improve our ability to predict CTD development and to identify the patients with better prognosis. The finding that only patients with IPAF/IPF evolve to a definite CTD (10/22) underscores that this patient group is clinically divergent and autoimmunity appears the driver of mechanistic process of the disease.

This study has several limitations: the retrospective and monocentric study design, the high rate of cases exclusion (343cases due to unavailability for review of autoimmune tests that are often performed by patients at their local hospital), the very small sample size of IPAF cases (*N* = 22) imbalanced compared to the controls groups [*N* = 43 non-IPAF/IPF positive serology, *N* = 295 non IPAF (IPF negative serology)]. Biases related to the observational retrospective design of this study spanning over a wide time period (2002–2016) are alleviated by the consecutive enrollment of patients, the reassessment of all IPF diagnosis based on ERS/ATS 2011 guidelines and the introduction in the multivariate survival analysis of the diagnostic time period (before/after the year 2011) as a dummy variable. However, concerns about the high dropout rate, a price we had to pay to achieve a highly accurately selected IPAF population with serology and rheumatology evaluation completed at our center, are only partially mitigated by the observation that the excluded cases compared to included cases had homogeneous clinical profile, although a slightly divergent prognosis.

## Conclusion

To the best of our knowledge, this is the first study showing that the presence of IPAF criteria in IPF has a major clinical impact correlating with the risk of evolution to full blown-CTD during follow-up (45.5% of IPAF/IPF patients develop CTD, none in the non-IPAF/IPF subgroup) and identifying a subgroup of patients with a clearly better prognosis (IPAF/IPF overall mortality adjusted HR 0.22).

Future prospective and larger studies will help to better define IPAF diagnostic criteria and their utility to identify in IPF specific subgroups with different prognosis and treatment response.

## Data availability statement

The original contributions presented in the study are included in the article/**Supplementary material**, further inquiries can be directed to the corresponding author.

## Ethics statement

This study was approved by the Comitato Etico di area vasta ROMagna, Italy (CEROM approval: protocol number 30/2020 I.5/284). Written informed consent [from the patients/participants OR patients/participants legal guardian/next of kin] was not required to participate in this study in accordance with the national legislation and the institutional requirements.

## Author contributions

ST, CR, SPu, SPi, AW, JR, MB, and VP: conception and design. ST, SPu, CR, MB, AW, JR, AD, SPi, FG, FL, ER, VL, MC, and VP: acquisition, analysis or interpretation, and drafting the manuscript for important intellectual content. VP had full access to all of the data in the study and takes responsibility for the integrity of the data and the accuracy of the data analysis. All authors contributed to the article and approved the submitted version.

## Conflict of interest

ST declares speaker’s fee from Boehringer-Ingelheim, Roche, Erbe, PulmoniX; and VP declares speaker’s fees from Boehringer-inghelhem, Erbe, Ambu, and Roche.

The remaining authors declare that the research was conducted in the absence of any commercial or financial relationships that could be construed as a potential conflict of interest.

## Publisher’s note

All claims expressed in this article are solely those of the authors and do not necessarily represent those of their affiliated organizations, or those of the publisher, the editors and the reviewers. Any product that may be evaluated in this article, or claim that may be made by its manufacturer, is not guaranteed or endorsed by the publisher.

## Abbreviations

ATS: American Thoracic Society
ERS: European Thoracic Society
IPAF: Interstitial pneumonia with autoimmune features
IIP: Idiopathic interstitial pneumonia
CTD: Connective tissue disease
NSIP: Non-specific interstitial pneumonia
IPF: Idiopathic pulmonary fibrosis
UIP: Usual interstitial pneumonia
NSIP/OP: Non-specific interstitial pneumonia/organizing pneumonia
ANA: Anti-nuclear antibodies
ANCA: Anti-neutrophil cytoplasmic antibody
ENA: Extractable Nuclear Antigen
CCP: Cyclic citrullinated peptide
RF: Rheumatoid factor
