# Supplementary material for: Clinical implications of interstitial pneumonia with autoimmune features diagnostic criteria in idiopathic pulmonary fibrosis: A case control study
Source: Front Med (Lausanne). 2023 Feb 16;10:1087485. doi: 10.3389/fmed.2023.1087485 (PMC9978138; doi:10.3389/fmed.2023.1087485)

IPAF Supplement

Index

Data collection and multidisciplinary evaluation of cases…………………………………………..2

Figure S1 Enrolment flow chart ……………………………………………………………………………...4

Table S1 Clinical characteristics of included and excluded cases ………………………………5

Table S2 Survival comparison between included and exclude cases ………………………..6

Figure S2 KM curve for included and excluded cases……………………………………………….7

Data collection and multidisciplinary evaluation of cases

Data extracted included: demographics information (age, gender, region of origin); clinical information (familiar history, smoking status and PY, environmental exposure, clinical onset, symptoms, mMRC, clinical signs); diagnosis information (date of first HRTC, histological results if available, BAL lymphocytosis level if available,); comorbidities at baseline and during follow-up (with special attention to emphysema, COPD, cancer and pulmonary hypertension); functional status at baseline (BMI, FVC ml, FVC %, FEV1 ml, FEV1 %, DLCO ml/mmHg/min, DLCO %, Oxygen use at rest/during exercise, walking distance at 6MWT) and during follow-up (FVC ml, FVC %, DLCO ml/mmHg/min, DLCO %). Date of last known follow-up, date of death or lung transplant. Drug treatment (antifibrotics, immunosuppressive treatment, steroid, warfarin, other).

Comorbidities were defined as one or more additional medical condition occurring with the ILD including but not limited to diabetes, myocardial infarction, cardiac heart failure, cardiovascular or cerebrovascular disease, pulmonary hypertension, dementia, chronic obstructive pulmonary disease, emphysema, gastrointestinal or ulcer bleeding, liver disease, kidney disease, gastroesophageal reflux, solid tumors, leukemia and lymphomas, AIDS, depression, sleep disorders. Sistolic Pulmonary Arterial Pressure was estimated using echocardiography.

The multidisciplinary evaluation was performed by pulmonary physicians, one chest radiologist (SP) and when required the pathologist (AD). The local rheumatologist (FG) was involved in the discussion of all cases suspected of having CTD-ILD or IPAF. The physicians were looking for the presence of each symptom and/or sign included in the clinical domain of IPAF criteria or suggestive of specific CTDs, as well as the presence of smoking habit, environmental exposure, disease duration, onset.

Autoimmunity test performed at our centre as part of the initial diagnostic work-up of

uncharacterized ILD and collected for all patients included: anti-TPO and anti-TG test, ANA, RF, anti-CCP, ANCA/MPO, ANCA/PR3, anti-RO/SSA, anti-LA/SSB, anti-RNP, anti-Smith antibody, anti-Scl-70 antibody and anti-ds/DNA. Myositis-specific antibodies were requested in selected cases based on clinical findings. ANA testing were performed using the indirect immunofluorescence method.

Data were collected with prospective intention following a standardized follow up protocol and that included: PFTs exams at baseline and then every 6 months; serologic test including autoimmunity test (auto-antibody anti-TPO and anti-TG test correlated to ANA, RF, anti-CCP, ANCA/MPO, ANCA/PR3, myositis-specific antibodies, anti-RO/SSA, anti-LA/SSB, anti-RNP, anti-Smith antibody, anti-Scl-70 antibody and anti-ds/DNA) at baseline and then annually; HRCT at baseline and every 12 months.

Comorbidities were also evaluated: reflux diagnosis was based on indirect signs revealed in the laryngoscopy or on pHmetry. Pulmonary hypertension diagnosis was not defined on the basis of right heart catheterism, but we estimated the PH likelihood using a cut off value of PAP 35 mmHg estimated on ecocardiography.

IPAF criteria for inclusions followed the ATS/ERS 2015 statement. We were particularly meticulous in: 1) excluding all cases without both serologic panel and rheumatology evaluation performed at our center; 2) excluding simple arthralgias and including only patients with documented inflammatory arthropathy that required one of swelling, tenderness, erythema, limitation in movement (including morning stiffness) or anatomic distortion; 3) excluding low levels of homogeneous or speckled ANA and RF and including only cases with ANA homogeneous or speckled ≥ 1/320 and/or RF ≥ 2 x UNL. 4) We were really rigorous in the definition of unexplained intrinsic airway disease and pulmonary vasculopathy. Unexplained airway disease was defined by the presence of follicular bronchiolitis or constrictive bronchiolitis by histopathology.

Neither low FEV1/FVC ratio, nor HRCT features of mosaic attenuation, air trapping or bronchiectasis were considered sufficient to define the presence of multicompartment criteria in IPF. The lung remodeling observed in IPF can cause pulmonary hypertension (PH), therefore neither the presence of PAPs ≥ 35mmHg measured at echocardiography, nor the dipropionate low DLco/FVC ratio (≥1.6) were considered adequate to define the presence of unexplained pulmonary vasculopathy suggestive of CTD.

Figure S1 Enrolment flow chart

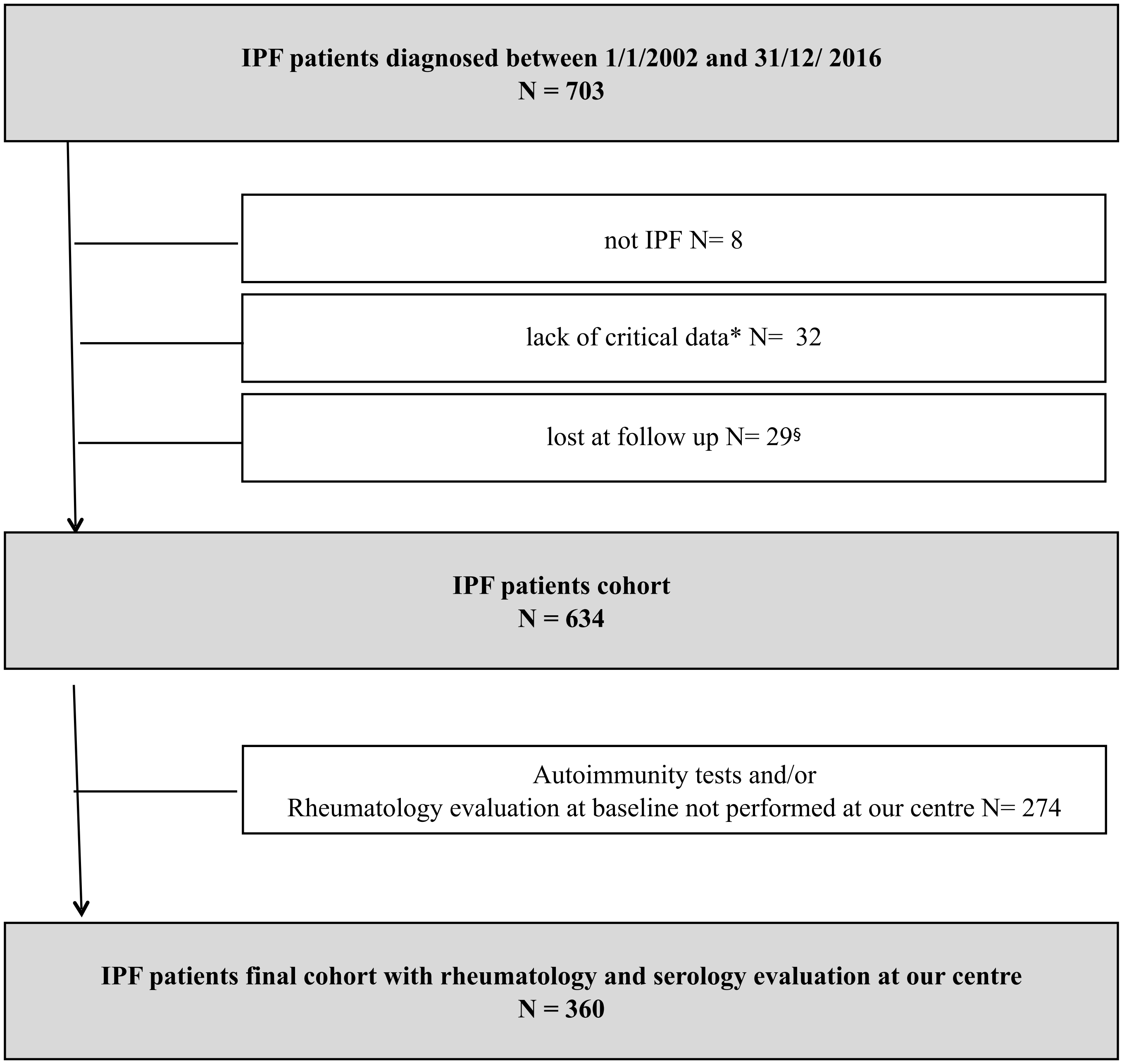

---

* critical data for IPF diagnosis were considered: HRCT, biopsy results, pulmonary function tests and clinical history at baseline available for revision.

§ Patients without follow-up evaluation (at least 3 months from baseline) and/or available vital status assessment were considered lost and excluded from the present study.

Table S1 Clinical characteristics of included and excluded cases

	Excluded	Included	p-value
N	N=274	N=360	
age, mean (SD)	66.80 (8.75)	66.59 (8.44)	0.76
Sex, male	194 (70.8%)	268 (74.4%)	0.31
eversmoke	182 (67.9%)	264 (74.2%)	0.087
comorbidities	233 (85.0%)	307 (85.3%)	0.93
number of comorbidities , mean (SD)	1.61 (1.18)	1.53 (1.06)	0.37
**lung cancer**	**18 (6.6%)**	**41 (11.4%)**	**0.039**
%FVC, mean (SD)	76.72 (18.68)	77.64 (19.62)	0.55
%DLco , mean (SD)	50.55 (16.68)	49.76 (16.66)	0.56

Table S2 Survival comparison between included and exclude cases

	Univariate Analysis		Multivariate Analysis	
	HR (95% CI)	p value	HR (95% CI)	p value
Age	1.02 (1.01-1.04)	**0.0005**	1.02 (1.01-1.04)	**<0.0001**
Sex Male	1.06 (0.65-1.26)	0.55	1.14 (0.88-1.49)	0.31
Smoking history	1.24 (0.98-1.57)	0.76	-	-
Comorbidities (yes/no)	0.92 (0.69-1.23)	0.57	-	-
Lung cancer (yes/no)	1.88 (1.39-2.56)	**0.0002**	2.10 (1.52-2.88)	**<0.0001**
Pulmonary Function				
% pred FVC	0.97 (0.97-0.98)	**<0.0001**	0.99 (0.98-0.99)	**<0.0001**
% pred DLco	0.95 (0.95-0.96)	**<0.0001**	0.96 (0.95-0.97)	**<0.0001**
Included cases	1.32 (1.07-1.63)	**0.011**	1.39 (1.11-1.73)	**0.003**

Figure S2 KM curve for included and excluded cases

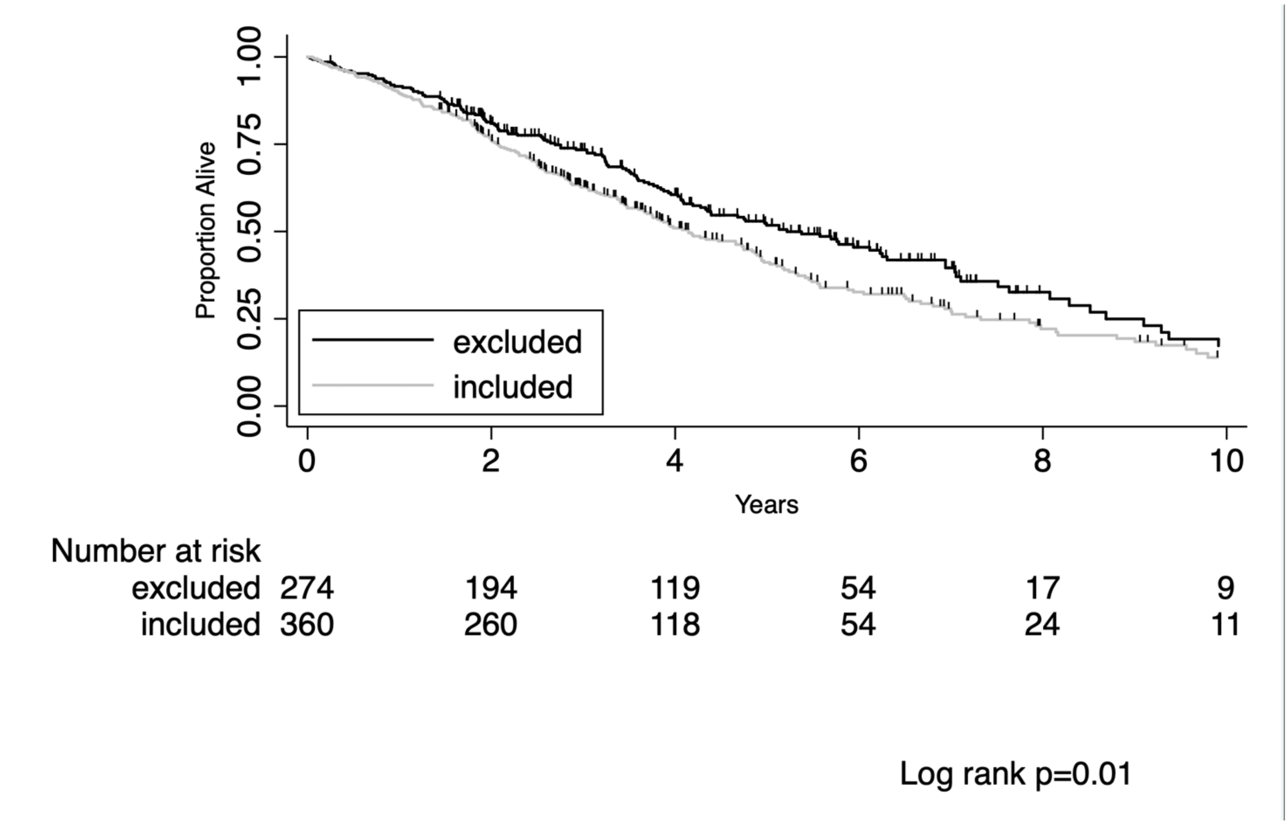

Supplement: Supplementary file 1 [file Data_Sheet_1.PDF]
